# Supplementary material for: Comparative Genome Analysis of Polar Mesorhizobium sp. PAMC28654 to Gain Insight into Tolerance to Salinity and Trace Element Stress
Source: Microorganisms. 2024 Jan 7;12(1):120. doi: 10.3390/microorganisms12010120 (PMC10820077; doi:10.3390/microorganisms12010120)
Supplement: Supplementary file 1 [file microorganisms-12-00120-s001.zip › microorganisms-2722535-supplementary.pdf]

Supplementary Material
for

Comparative genome analysis of polar *Mesorhizobium* sp. PAMC28654 to gain insight into tolerance for salinity and trace element stress

Anamika Khanal[1,2], So-Ra Han[1,2,3], Jun Hyuck Lee[4], and Tae-Jin Oh[1,2,3,5*]

[1] Genome-based Bio-IT Convergence Institute, Asan 31460, South Korea; anamika.khanal@gmail.com (A.Khanal)

[2] Bio Big Data-based Chungnam Smart Clean Research Leader Training Program, SunMoon University, Asan 31460, South Korea; 553sora@hanmail.net (S.-R. Han)

[3] Department of Life Science and Biochemical Engineering, Graduate School, SunMoon University, Asan 31460, South Korea

[4] Research Unit of Cryogenic Novel Materials, Korea Polar Research Institute, Incheon 21990, South Korea; junhyucklee@kopri.re.kr (J.H.Lee)

[5] Department of Pharmaceutical Engineering and Biotechnology, SunMoon University, Asan 31460, South Korea

*** Correspondence**: Prof. Tae-Jin Oh

Department of Pharmaceutical Engineering and Biotechnology, SunMoon University, Asan 31460, South Korea. E-mail address: tjoh3782@sunmoon.ac.kr (T.-J.Oh).

Running title: Genome analysis of *Mesorhizobium* strain for abiotic stresses.

Keywords: Cold adaptation; comparative genomics; exopolysaccharide; heavy metal; *Mesorhizobium* sp. PAMC28654; salinity.

Legends for Supplementary Tables and Supplementary Figures

Supplementary Figure S1. Putative 3D modelling structure of nitrate reductase (Nar) protein of *Mesorhizobium* sp. PAMC28654. (A) NarG, α-subunit; (B) NarH, β subunit; and (C) NarI, γ subunit. The putative 3D images were generated in Jsmol colored by rainbow from N to C terminals.

Supplementary Figure S2. *Mesorhizobium* sp. PAMC28654 showing nitrate reduction. (A) *Mesorhizobium* sp. PAMC28654 culture in R2A agar. (B) Nitrate reduction at 15°C by *Mesorhizobium* sp. PAMC28654. lane 1, Abiotic control containing nitrate media; 2, Abiotic control containing nitrate media with reagent A and reagent B; 3, Abiotic control containing nitrate media with reagent A, reagent B, and addition of zinc; 4, Positive control containing nitrate media using *E. coli* without reagent A and reagent B; 5, Positive control containing nitrate media using *E. coli* with reagent A and reagent B; 6, *Mesorhizoium* sp. PAMC28654 without reagent A and reagent B; 7, 8, and 9, *Mesorhizoium* sp. PAMC28654 with reagent A and reagent B. Experiments were performed in triplicates. (C) Nitrate reduction at 15°C by *Mesorhizobium* sp. PAMC28654. lane 1, Abiotic control containing nitrate media; 2, Abiotic control containing nitrate media with reagent A and reagent B; 3, Abiotic control containing nitrate media with reagent A, reagent B, and addition of zinc; 4, Positive control containing nitrate media using *E. coli* without reagent A and reagent B; 5, Positive control containing nitrate media using *E. coli* with reagent A and reagent B; 6, *Mesorhizoium* sp. PAMC28654 without reagent A and reagent B; 7, 8, and 9, *Mesorhizoium* sp. PAMC28654 with reagent A and reagent B. Experiments were performed in triplicates.

Supplementary Figure S3. Exopolysachharide (crude) produced by *Mesorhizobium* sp. PAMC28654 at 15°C and 25°C, respectively.

Supplementary Table S1. Genomic information of available genome of *Mesorhizobium* strains from NCBI database.

Supplementary Table S2. Genomic information of available genome of *Mesorhizobium* strains from NCBI database.

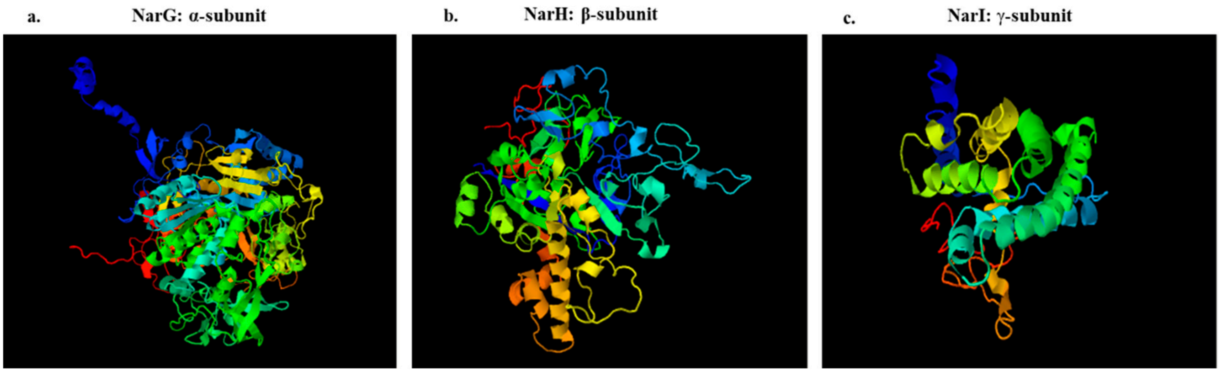

Supplementary Figure S1. Putative 3D modelling structure of nitrate reductase (Nar) protein of *Mesorhizobium* sp. PAMC28654. (A) NarG, α-subunit; (B) NarH, β subunit; and (C) NarI, γ subunit. The putative 3D images were generated in Jsmol colored by rainbow from N to C terminals.

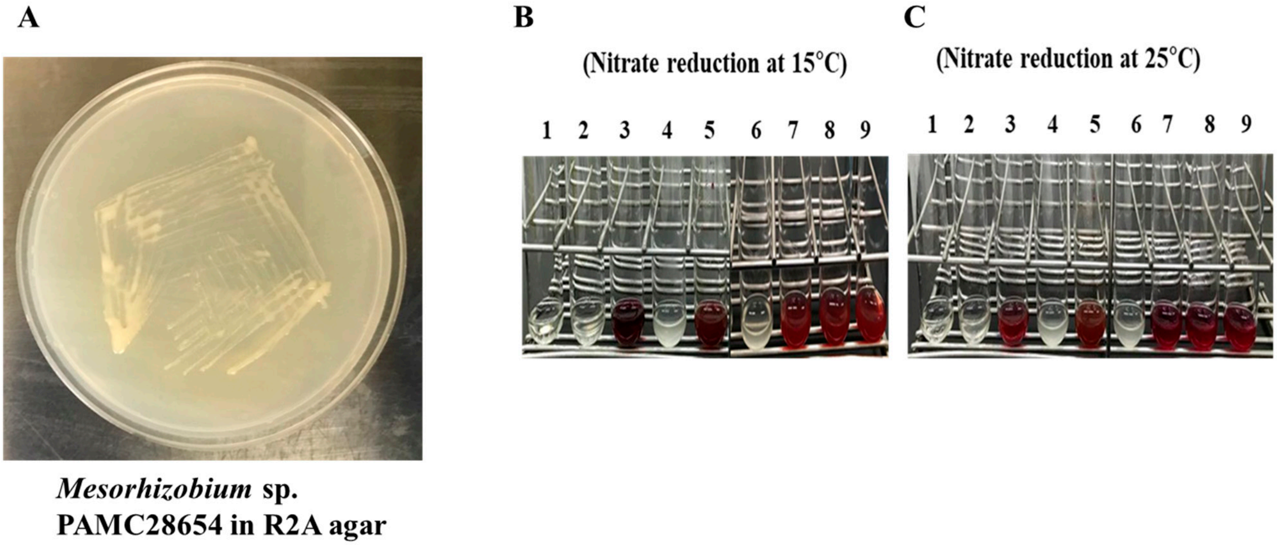

Supplementary Figure S2. *Mesorhizobium* sp. PAMC28654 showing nitrate reduction. (A) *Mesorhizobium* sp. PAMC28654 culture in R2A agar. (B) Nitrate reduction at 15°C by *Mesorhizobium* sp. PAMC28654. lane 1, Abiotic control containing nitrate media; 2, Abiotic control containing nitrate media with reagent A and reagent B; 3, Abiotic control containing nitrate media with reagent A, reagent B, and addition of zinc; 4, Positive control containing nitrate media with *E. coli* without reagent A and reagent B; 5, Positive control containing nitrate media using *E. coli* with reagent A and reagent B; 6, *Mesorhizoium* sp. PAMC28654 without reagent A and reagent B; 7, 8, and 9, *Mesorhizoium* sp. PAMC28654 with reagent A and reagent B. Experiments were performed in triplicates. (C) Nitrate reduction at 15°C by *Mesorhizobium* sp. PAMC28654. lane 1, Abiotic control containing nitrate media; 2, Abiotic control containing nitrate media with reagent A and reagent B; 3, Abiotic control containing nitrate media with reagent A, reagent B, and addition of zinc; 4, Positive control containing nitrate media using *E. coli* without reagent A and reagent B; 5, Positive control containing nitrate media using *E. coli* with reagent A and reagent B; 6, *Mesorhizoium* sp. PAMC28654 without reagent A and reagent B; 7, 8, and 9, *Mesorhizoium* sp. PAMC28654 with reagent A and reagent B. Experiments were performed in triplicates.

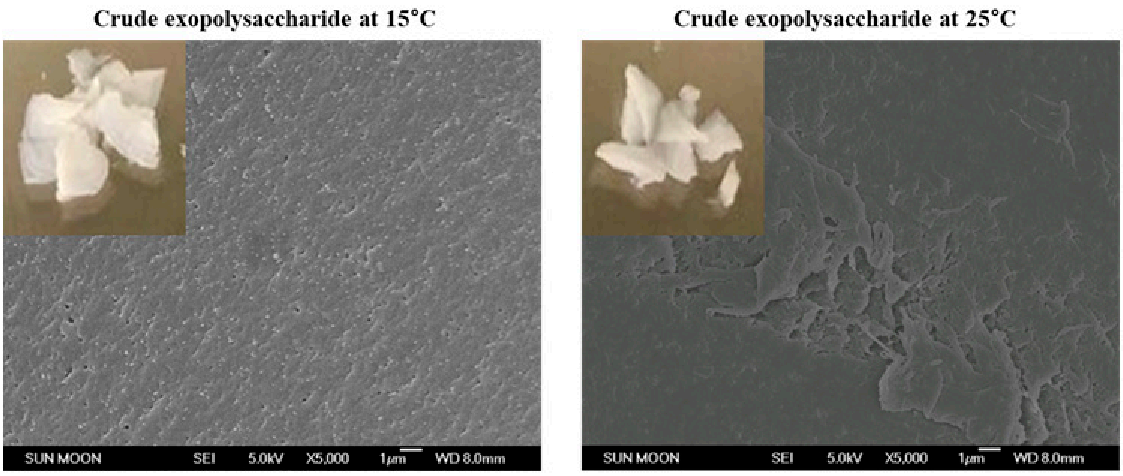

Supplementary Figure S3. Exopolysachharide (crude) produced by *Mesorhizobium* sp. PAMC28654 at 15°C and 25°C, respectively.

Supplementary Table S1. Genomic information of available genome of *Mesorhizobium* strains from NCBI database.

Organism	Strain	Isolate information							
		Isolation source	Isolation country	Geographic location	Host	Sample type	Collection date	Accession number of genome sequence	Reference
Mesorhizobium sp.	PAMC28654	Soil	Uganda	Uganda	N/A	N/A	2019	NZ_CP085135.1	This study
Mesorhizobium sp.	INR15	Soil	N/A	Antarctica, Robinson ridge	N/A	Pure culture	2017	NZ_CP045496.1	[32]
Mesorhizobium sp.	NBSH29	Soil	N/A	Antarctica, Herring Island	N/A	Pure culture	2017	NZ_CP045492.1	[32]
Mesorhizobium loti	R88b	N/A	New Zealand	New Zealand	*Lotus corniculatus*	N/A	1993	NZ_CP033367.1	[33]
Mesorhizobium sp.	AA22	Root nodule	Ethopia	Ethopia	*Astragalus pelecinus* L.	Cell culture	2014	NZ_CP048406.1	[40]
Mesorhizobium sp.	113-1-2	N/A	Japan	Japan, Tottori, Kotoura, Kasechi river	*Lotus japonicus*	N/A	2016	NZ_AP023242.1	[49]
Mesorhizobium sp.	113-3-3	N/A	Japan	Japan, Tottori, Kotoura, Kasechi river	*Lotus japonicus*	N/A	2016	NZ_AP023243.1	[49]
Mesorhizobium sp.	113-3-9	N/A	Japan	Japan, Tottori, Kotoura, Kasechi river	*Lotus japonicus*	N/A	2016	NZ_AP023246.1	[49]
Mesorhizobium sp.	131-2-1	N/A	Japan	Japan, Aomori, Okidate, Aomori port	*Lotus japonicus*	N/A	2016	NZ_AP023247.1	[49]
Mesorhizobium sp.	131-2-5	N/A	Japan	Japan, Aomori, Okidate,	*Lotus japonicus*	N/A	2016		[49]

				Aomori port				NZ_AP023249.1	
Mesorhizobium sp.	131-3-5	N/A	Japan	Japan, Aomori, Okidate, Aomori port	*Lotus japonicus*	N/A	2016	NZ_AP023254.1	[49]
Mesorhizobium sp.	L-2-11	Root nodule	Japan	Japan, Okinawa, Miyakojima, Higashihenna cape	*Lotus japonicus*	N/A	2016	NZ_AP023263.1	[49]
Mesorhizobium sp.	L-8-10	Root nodule	Japan	Japan, Okinawa, Miyakojima, Higashihenna cape	*Lotus japonicus*	N/A	2016	NZ_AP023263.1	[49]
Mesorhizobium sp.	L-8-3	Root nodule	Japan	Japan, Okinawa, Miyakojima, Higashihenna cape	*Lotus japonicus*	N/A	2016	NZ_AP023262.1	[49]
Mesorhizobium sp.	8	Farmland	China	China, Dezhou	N/A	N/A	2017	NZ_CP040914.1	Unpublished
Mesorhizobium sp.	AR02	Root nodule	Canada	Canada, Nort west territori es, Daring lake region	*Oxytropis* sp.	Cell culture	2019	NZ_CP080531.1	[50]
Mesorhizobium sp.	AR07	Root nodule	Canada	Canada, Nort west territori es, Daring lake region	*Oxytropis* sp.	Cell culture	2019	NZ_CP080525.1	[50]
Mesorhizobium sp.	AR10	Root nodule	Canada	Canada, Nort west territori es, Daring lake region	*Astragalus* sp.	Cell culture	2019	NZ_CP080524.1	[50]
Mesorhizobium sp.	B1-1-8	Soil	Australia: WA, Ba dgingarra	Australia	N/A	Cell culture	2019	NZ_CP083956.1	Unpublished
Mesorhizobium sp.	B2-1-1	Soil	Australia: WA, Ba dgingarra	Australia	N/A	Cell culture	2019	NZ_CP083954.1	Unpublished

Mesorhizobium sp.	B2-1-8	Soil	Australia: WA, Ba dgingarra	Australia	N/A	Cell culture	2019	NZ_CP083952.1	Unpublished
Mesorhizobium sp.	B2-8-5	Soil	Australia: WA, Ba dgingarra	Australia	N/A	Cell culture	2019	NZ_CP083951.1	Unpublished
Mesorhizobium sp.	B4-1-4	Soil	Australia: WA, Ba dgingarra	Australia	N/A	Cell culture	2019	NZ_CP083950.1	Unpublished
Mesorhizobium sp.	J8	Soil	Japan	Japan, Saga	*Glycyrrhiza uralensis*	N/A	2014	NZ_ AP024109.1	[51]
Mesorhizobium sp.	M1B.F.Ca.ET.045.0 4.1.1	Root nodule	Ethopia	Ethopia, Amhara, North Gondar	*Cicer arietinum*	Bacterial culture	2014	NZ_CP034448.1	[52]
Mesorhizobium sp.	M1D.F.Ca.ET.043. 01.1.1	Root nodule	Ethopia	Ethopia, Amhara, North Gondar	*Cicer arietinum*	Bacterial culture	2014	NZ_CP034444.1	[52]
Mesorhizobium sp.	M1E.F.Ca.ET.045.0 2.1.1	Root nodule	Ethopia	Ethopia, Amhara, North Gondar	*Cicer arietinum*	Bacterial culture	2014	NZ_CP034447.1	[52]
Mesorhizobium sp.	M2A.F.Ca.ET.043. 02.1.1	Root nodule	Ethopia	Ethopia, Amhara, North Gondar	*Cicer arietinum*	Bacterial culture	2014	NZ_CP034445.1	[52]
Mesorhizobium sp.	M2A.F.Ca.ET.043. 05.1.1	Root nodule	Ethopia	Ethopia, Amhara, North Gondar	*Cicer arietinum*	Bacterial culture	2014	NZ_CP034446.1	[52]
Mesorhizobium sp.	M2A.F.Ca.ET.046. 03.2.1	Root nodule	Ethopia	Ethopia, Amhara, North Gondar	*Cicer arietinum*	Bacterial culture	2014	NZ_CP034449.1	[52]
Mesorhizobium sp.	M3A.F.Ca.ET.080. 04.2.1	Root nodule	Ethopia	Ethopia, Amhara, North Gondar	*Cicer arietinum*	Bacterial culture	2014	NZ_CP034451.1	[52]
Mesorhizobium sp.	M9A.F.Ca.ET.002. 03.1.2	Root nodule	Ethopia	Ethopia, Amhara, North Gondar	*Cicer arietinum*	Bacterial culture	2014	NZ_CP034443.1	[52]

Mesorhizobium sp.	NZP2077	Lotus corniculatus nodule	New Zealand	New Zealand, Kalkohe	N/A	Cell culture	1991	NZ_CP051293.1	Unpublished
Mesorhizobium sp.	NZP2077	N/A	New Zealand	New Zealand, Kalkohe	N/A	Cell culture	1991	NZ_CP033362.1	Unpublished
Mesorhizobium sp.	NZP2234	N/A	USA	USA	*Lotus corniculatus*	Cell culture	1982	NZ_CP033364.1	Unpublished
Mesorhizobium sp.	NZP2298	N/A	Canada	Canada	*Lotus corniculatus*	Cell culture	1889	NZ_CP033365.1	Unpublished
Mesorhizobium sp.	Pch-S	Micro-algae	South Ko rea	South Korea	*Paulinella*	Cell culture	2016	NZ_CP029562.1	Unpublished
Mesorhizobium sp.	WSM1497	Root nodule	Greece	Greece	*Biserrula pelecinus* L.	Cell culture	1995	NZ_CP021070.1	Unpublished
Mesorhizobium sp.	WSM4904	N/A	Australia	Australia, WA, Oasis Farms	*Cicer arietinum ka buli*	Cell culture	2017	NZ_CP121354.1	Unpublished
Mesorhizobium sp.	WSM4906	N/A	Australia	Australia, WA, Oasis Farms	*Cicer arietinum ka buli*	Cell culture	2017	NZ_CP121355.1	Unpublished
Mesorhizobium ciceri	R30	Radical nodules from chickpeas	USA	USA, Maryland	*Cicer arietinum* L.	Cell culture	N/A	NZ_CP088147.1	[53]
Mesorhizobium ciceri	CC1192	N/A	Israel	Israel	*Cicer arietinum*	Pure sample	1977	NZ_CP015062.1	[54]
Mesorhizobium ciceri b iovar biserrulae	WSM1284	Root nodule	Italy	Italy, Sardinia, Siniscola	*Astragalus pelecin us*	Pure sample	1993	NZ_CP015064.1	[55]
Mesorhizobium ciceri b iovar biserrulae	WSM1271	Howleson	Italy	Italy	*Biserrula pelecinus* L.	N/A	1993	NZ_CP002447.1	Unpublished
Mesorhizobium loti	SU343	N/A	Australia	Australia, NSW	*Lotus* sp.	Cell culture	1971	NZ_CP033368.1	Unpublished
Mesorhizobium loti	NZP2042	N/A	New Zeal	New Zealand, Palmerston	*Lotus* sp.	Cell culture	1971	NZ_CP033334.1	Unpublished

			and	North					
Mesorhizobium loti	582	Root nodule	Russia	Russia, Kamchatka	*Oxytropis kamtschatica*	Cell culture	2018	NZ_CP050293.1	[56,57]
Mesorhizobium huakuii	583	Root nodule	Russia	Russia, Kamchatka	*Oxytropis kamtschatica*	Cell culture	2018	NZ_CP050296.1	[56,57]
Mesorhizobium loti	NZP2037	N/A	New Zealand	New Zealand, Palmerston North	*Lotus*	Cell culture	1961	NZ_CP016079.1	Unpublished
Mesorhizobium loti	R88b	N/A	New Zealand	New Zealand	*Lotus corniculatus*	N/A	1993	NZ_CP033367.1	Unpublished
Mesorhizobium japonicum	R7Astar	N/A	New Zealand	New Zealand, Dunedin	N/A	N/A	2007	NZ_CP051773.1	Unpublished
Mesorhizobium japonicum	R7AstarV2	N/A	New Zealand	New Zealand, Dunedin	N/A	Cell culture	2014	NZ_CP052769.1	Unpublished
Mesorhizobium japonicum	R7ANSstar	N/A	New Zealand	New Zealand, Dunedin	N/A	Cell culture	2014	NZ_CP052770.1	Unpublished
Mesorhizobium japonicum^{T}	MAFF 303099	N/A	N/A	N/A	N/A	N/A	N/A	NZ_BA000012.4	[58]
Mesorhizobium japonicum	R7A	N/A	New Zealand	Lammermoor, Otago NZ	*Lotus*	N/A	N/A	NZ_CP051772.1	Unpublished
Mesorhizobium japonicum	R7A	Soil, root nodule	New Zealand	Lammermoor, Otago NZ	*Lotus*	N/A	N/A	NZ_CP033366.1	Unpublished
Mesorhizobium amorphae	CCNWGS0123	N/A	China	China, Gansu	*Robinia pseudoacacia*	Cell culture	2006	NZ_CP015318.1	Unpublished
Mesorhizobium australicum^{T}	WSM2073	Nandasena	Australia	Western Australia, antonio's farm, Antonio Rd.	*Biserrula pelecinus* L.	Type strain	N/A	NZ_CP003358.1	Unpublished
Mesorhizobium opportunistum	WSM1558	N/A	Italy	Italy, Ozieri, Province of sassari, Sardinia	*Astragalus pelecinus*	Cell culture	1995	NZ_CP097252.1	Unpublished

Mesorhizobium opportunistum[T]	WSM2075	Nandasena	Australia	Australia, Antonio's farm, Antonio Rd, Northam,	*Biserrula pelecinus* L	Type strain	N/A	NZ_CP002279.1	Unpublished
Mesorhizobium erdmanii	NZP2014	N/A	New Zealand	New Zealand, Palmerston North	*Lotus* sp.	Cell culture	1971	NZ_CP033361.1	Unpublished
Mesorhizobium jarvisii[T]	ATCC 33669	N/A	New Zealand	New Zealand, Palmerston North	*Lotus corniculatus*	Cell culture	1976	NZ_CP033507.1	Unpublished

N/A: Not Available

Supplementary Table S2. Genomic information of available genome of *Mesorhizobium* strains from NCBI database.

Strain name	GC %	Genome total length	No of chromosome	No of plasmid	Total number of proteins	rRNA genes	tRNA genes
Mesorhizobium sp. PAMC28654	62.20	6.70	1	0	6.17	6	54
Mesorhizobium sp. INR15	61.96	7.45	1	4	7.00	6	52
Mesorhizobium sp. NBSH29	58.96	4.00	1	3	3.81	3	44
Mesorhizobium loti R88b	62.40	7.20	1	0	6.71	6	51
Mesorhizobium sp. AA22	61.90	6.61	1	0	5.87	6	58
Mesorhizobium sp. 113-1-2	62.80	7.93	1	0	7.34	6	53
M*esorhizobium* sp. 113-3-3	62.82	7.73	1	2	7.13	6	52
Mesorhizobium sp. 113-3-9	62.90	7.59	1	1	7.03	6	52
Mesorhizobium sp. 131-2-1	63.56	7.10	1	1	6.57	6	53
Mesorhizobium sp. 131-2-5	62.49	7.99	1	4	7.37	6	53
Mesorhizobium sp. 131-3-5	62.68	7.57	1	2	7.04	6	54
Mesorhizobium sp. L-2-11	62.23	7.41	1	4	6.55	6	59
Mesorhizobium sp. L-8-10	64.20	8.45	1	0	7.57	6	52
Mesorhizobium sp. L-8-3	64.20	8.44	1	0	7.48	6	52
Mesorhizobium sp. 8	65.20	4.81	1	0	4.52	3	47
Mesorhizobium sp. AR02	62.32	8.90	1	3	8.18	6	52
Mesorhizobium sp. AR07	62.05	8.73	1	3	7.95	6	53
Mesorhizobium sp. AR10	62.03	6.87	1	1	6.35	3	50

Mesorhizobium sp. B1-1-8	63.24	6.18	1	1	5.83	3	49
Mesorhizobium sp. B2-1-1	63.11	6.18	1	1	5.82	6	48
Mesorhizobium sp. B2-1-8	62.64	6.87	1	1	6.44	6	54
Mesorhizobium sp. B2-8-5	63.80	6.46	1	0	6.46	6	54
Mesorhizobium sp. B4-1-4	62.50	6.27	1	0	5.75	6	50
Mesorhizobium sp. J8	63.80	6.69	1	0	6.27	6	53
Mesorhizobium sp. M1B.F.Ca.ET.045.04.1.1	63.10	7.77	1	0	7.08	7	54
Mesorhizobium sp. M1D.F.Ca.ET.043.01.1.1	63.40	7.12	1	0	6.48	6	53
Mesorhizobium sp. M1E.F.Ca.ET.045.02.1.1	63.20	7.34	1	0	6.74	6	52
Mesorhizobium sp. M2A.F.Ca.ET.043.02.1.1	63.60	6.70	1	0	6.12	6	55
Mesorhizobium sp. M2A.F.Ca.ET.043.05.1.1	63.60	6.74	1	0	6.14	3	48
Mesorhizobium sp. M2A.F.Ca.ET.046.03.2.1	63.20	7.37	1	0	6.67	6	55
Mesorhizobium sp. M3A.F.Ca.ET.080.04.2.1	63.30	6.16	1	0	5.56	3	45
Mesorhizobium sp. M9A.F.Ca.ET.002.03.1.2	62.50	6.41	1	0	5.79	3	53
Mesorhizobium sp. NZP2077	62.26	7.68	1	1	7.12	6	54
Mesorhizobium sp. NZP2077	62.44	7.19	1	1	6.73	6	54
Mesorhizobium sp. NZP2234	63.10	6.74	1	0	6.30	6	52

Mesorhizobium sp. NZP2298	62.80	7.33	1	0	6.77	6	56
Mesorhizobium sp. Pch-S	62.20	6.61	1	0	6.09	6	49
Mesorhizobium sp. WSM1497	62.41	7.19	1	1	6.56	6	52
Mesorhizobium sp. WSM4904	N/A	6.65	1	0	6.22	3	47
Mesorhizobium sp. WSM4906	N/A	6.68	1	0	6.24	3	49
Mesorhizobium ciceri	62.51	6.94	1	1	6.47	6	52
Mesorhizobium ciceri	62.49	6.94	1	1	6.49	6	54
Mesorhizobium ciceri biovar biserrulae	62.51	6.88	1	1	6.34	6	52
Mesorhizobium ciceri biovar biserrulae WSM1271	62.57	6.69	1	1	6.23	6	52
Mesorhizobium loti	62.89	7.20	1	2	6.80	6	52
Mesorhizobium loti	63.10	6.87	1	0	6.44	3	47
Mesorhizobium loti	62.46	8.33	1	2	7.35	6	55
Mesorhizobium loti NZP2037	62.74	7.48	1	1	6.99	6	52
Mesorhizobium loti R88b	62.40	7.20	1	0	6.71	6	51
Mesorhizobium japonicum	62.90	6.53	1	0	6.07	6	51
Mesorhizobium japonicum	62.90	6.53	1	0	6.07	6	51
Mesorhizobium japonicum	63.20	6.02	1	0	5.69	6	51
Mesorhizobium japonicum MAFF 303099^{T}	62.46	7.59	1	2	7.05	6	52
Mesorhizobium japonicum R7A	62.90	6.53	1	0	6.07	6	51
Mesorhizobium japonicum R7A	62.90	6.52	1	0	6.07	6	51

Mesorhizobium amorphae CCNWGS0123	62.83	7.34	1	4	6.59	6	52
Mesorhizobium huakuii	62.50	8.44	1	3	7.22	6	51
Mesorhizobium australicum WSM2073[T]	62.80	6.20	1	0	5.76	6	53
*Mesorhizobium oppurtunistum*WSM1558	62.70	6.87	1	0	6.37	6	53
*Mesorhizobium op*purtunistumWSM2075[T]	62.90	6.88	1	0	6.49	6	53
Mesorhizobium erdmanii	62.90	6.60	1	0	6.12	6	53
Mesorhizobium jarvisii[T]	62.89	7.20	1	2	6.80	6	52

N/A: Not Available
